# Supplementary figures and images for: Assessment of combined serum sST2 and AFP levels in the diagnosis of hepatocellular carcinoma
Source: PeerJ. 2024 Oct 8;12:e18142. doi: 10.7717/peerj.18142 (PMC11639131; doi:10.7717/peerj.18142)

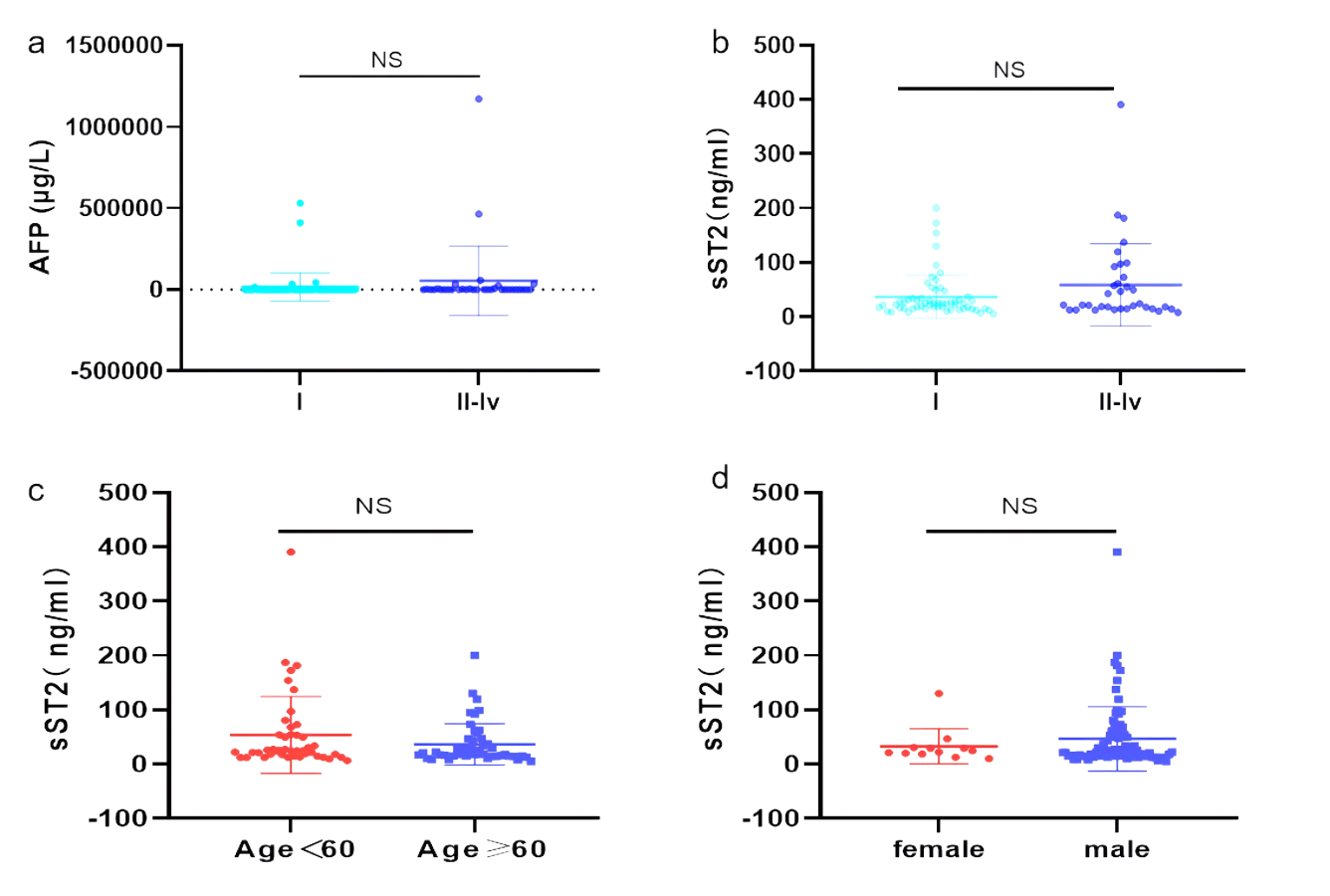

Supplement: Supplemental Information 2 — (a)The AFP levels between TNM stage I and II–IV in HCC group.(b) The sST2 levels between TNM stage I and II–IV in HCC group. (c) The sST2 levels between the patients aged ≥60y and aged <60y in HCC group. (d) The sST2 levels between male and female in HCC group. [file peerj-12-18142-s002.png]
